# Adapting the European Concerted Action on Congenital Anomalies and Twins (EUROCAT) Guide 1.5 for Use in Post‐Authorisation Safety Studies Using US Data

**DOI:** 10.1002/pds.70109

**Published:** 2025-02-15

**Authors:** Sarah Ruth Hoffman, Geetika Kalloo, Stephan Lanes, Aziza Jamal‐Allial, Todd Sponholtz, Corinne Brooks, Maria Guzman, Maria I. Van Rompay, Oluwadamilola Onasanya, Vincent J. Willey, Erica Foster, Nadia Messeh, Jill Layton, Dawn Ponist, Maryline L. E. Noan‐Lainé, Krista Schroeder

**Affiliations:** ^1^ Safety and Epidemiology Carelon Research Wilmington DE USA; ^2^ Eli Lilly & Company Indianapolis IN USA

**Keywords:** birth defects, claims database, congenital malformations, post‐authorisation safety study (PASS), post‐marketing requirement (PMR), pregnancy, safety

## Abstract

**Purpose:**

Many post‐authorization safety studies focus on congenital malformations and rely on diagnosis codes found in US data sources. However, no authoritative standards exist for identifying and classifying malformations in these data. To address this, we translated an existing public health surveillance guide, the European Concerted Action on Congenital Anomalies and Twins (EUROCAT), into an ICD‐10‐CM code list for use in studies using US administrative healthcare data. The EUROCAT guide was selected for its decisive major or minor classification of each code. However, translation was required for use in US data sources since EUROCAT utilizes ICD‐10‐BPA which differs from ICD‐10‐CM (the coding system commonly encountered in US data sources).

**Methods:**

We mapped EUROCAT to ICD‐10‐CM. For each code, manual review was conducted by two or more researchers, and major/minor classification was based on code descriptions since some codes differed between coding systems.

**Results:**

A final code list was created, containing 916 ICD‐10‐CM codes for 744 major and 172 minor malformations. The code list contains ICD‐10‐CM codes, their corresponding descriptions, their major or minor classification and disease category according to EUROCAT, and variables indicating anomalies caused by genetic or infectious diseases unlikely attributable to a medication.

**Conclusions:**

We adapted the EUROCAT Guide 1.5 into an ICD‐10‐CM code list for use in pregnancy studies using US data sources. This list includes new ICD‐10‐CM codes available in 2024. As new ICD‐10‐CM codes become available, or as the EUROCAT Guide is updated, further updates to this list will be needed.

**Summary**

- Studies of drug safety during pregnancy often focus on congenital malformations which are classified as major or minor.
- No authoritative list of major and minor congenital malformations was available for use in studies that rely on diagnosis codes found in US data sources.
- The EUROCAT provides guidance to clinicians to code and report malformations to agencies conducting public health surveillance in Europe.
- EUROCAT uses a different coding system than do US health insurance companies.
- We translated the EUROCAT coding into a code list for drug safety studies that rely on data from US health insurance claims.

## 1 Introduction

Observational safety studies in the post‐authorization setting are used to evaluate the safety of newly approved drugs when used during pregnancy [1]. Many of these studies focus on congenital malformations and rely on diagnosis codes found in US healthcare data. However, to date, no authoritative standards exist for identifying and classifying malformations in these data.

In contrast, multiple coding and reporting guidelines exist for clinicians reporting malformations to public health agencies [2, 3, 4]. One such standard is the European Concerted Action on Congenital Anomalies and Twins (EUROCAT). The EUROCAT is a surveillance system established in 1979, “to provide essential epidemiological information on congenital anomalies in Europe” [5]. This system provides comprehensive lists for both major and minor congenital malformations and their corresponding diagnosis codes according to the International Classification of Diseases 10th Revision (ICD‐10) [6] and British Paediatric Association (BPA) extension (ICD‐10‐BPA) [7].

While EUROCAT provides a comprehensive list of diagnosis codes for both major and minor malformations, this resource has not yet been formally adapted for use in the United States where ICD‐10‐Clinical Modification (ICD‐10‐CM) [8] codes are used. The ICD‐10‐CM coding system contains numerous codes that are not included in ICD‐10 (e.g., Q13.81 “Rieger's anomaly”). Additionally, EUROCAT utilizes BPA extension codes which do not consistently correspond to ICD‐10‐CM codes and do not appear in United States claims databases. Finally, the EUROCAT format is not readily useable as a code list which can be imported to statistical analysis software.

For these reasons, we endeavored to translate the EUROCAT Guide 1.5 ICD‐10‐BPA classification of major and minor malformations into an ICD‐10‐CM code list for use in post‐authorisation safety studies using US administrative claims data.

## 2 Methods

We downloaded the ICD‐10‐CM 2022 database from the US National Center for Health Statistics (NCHS) as a Microsoft Excel file [8]. The principal investigator classified as major all ICD‐10‐CM codes contained in the “All Anomalies” row and “ICD10‐BPA” column of EUROCAT 1.5 Section 3.3, and then classified as minor all ICD‐10‐CM codes matching those found in the “Excluded minor anomalies post‐2005” column of Section 3.3 and listed in Section 3.2. The ICD‐10‐CM 2024 database was subsequently downloaded from NCHS, new Q codes not appearing in the 2022 list (*n* = 38) were identified, and the above process was repeated to include them.

According to EUROCAT Section 3.3, ICD‐10 code Q33.6 “Hypoplasia and dysplasia of lung” is excluded from both the major and minor anomalies lists. Its corresponding ICD‐10‐CM code is Q33.6 “Congenital hypoplasia and dysplasia of lung.” We retained this code in the code list but did not assign it a major or minor classification, instead coding the major/minor field as missing (“.”). This allows research teams to choose whether to include the code and assign it a classification of their choosing (e.g., based on additional information from the medical record).

When major/minor classification could not be determined due to a BPA extension code that was more specific than ICD‐10‐CM, a toggle variable was created to allow for study team discretion to assign a major or minor classification. For example, EUROCAT classifies all ICD‐10 codes beginning with Q04 “Other congenital malformations of brain” as major except for ICD‐10‐BPA Q04.61 “Single congenital cerebral cyst.” Both ICD‐10 and ICD‐10‐CM contain code Q04.6 “Congenital cerebral cysts.” Without the BPA extension, there is no way to distinguish multiple congenital cerebral cysts (ICD‐10‐BPA Q04.60) from a minor single congenital cerebral cyst (ICD‐10‐BPA Q04.61). We classified ICD‐10‐CM Q04.6 “Congenital cerebral cysts” as major and included a value of “1” for the toggle variable “minorBPA” to indicate that this code may encompass some minor anomalies, in this case single congenital cerebral cysts. Using this toggle variable, the study team can change the code's classification from major to minor.

We created variables to indicate genetic and infectious anomalies that are often excluded from drug safety studies. In addition, disease categories were created to classify malformations by organ system consistent with EUROCAT.

A total of 17 major anomaly codes (Q27.1‐Q28.9) did not have an organ system classification in EUROCAT. Given that these codes are included under the “Congenital malformations of the circulatory system” section of both ICD‐10 and ICD‐10‐CM, we assigned them to the “Cardiovascular” category.

A team of scientists (*n* = 11) conducted a quality control investigation to ensure appropriate translation. Each reviewer was assigned between 80 and 90 ICD‐10‐CM codes to review. When codes and descriptions matched, the ICD‐10‐CM codes were given the major/minor classification appearing in EUROCAT. For non‐matching codes, manual review was conducted, and major/minor classification was based on matching descriptions between ICD‐10‐BPA (EUROCAT) and ICD‐10‐CM. When reviewers disagreed with the principal investigator's classification, a discussion was held between the reviewer and principal investigator until a consensus was reached.

During the quality control review, there were minor anomalies listed in EUROCAT Section 3.2 that did not have corresponding codes in ICD‐10 or ICD‐10‐BPA but did have codes available in ICD‐10‐CM. The ICD‐10‐CM codes were then re‐examined for descriptions matching the minor anomalies listed without codes in EUROCAT Section 3.2 to ensure that they were correctly classified as minor. For example, EUROCAT Section 3.2 lists “buried penis” as a minor anomaly without a corresponding code as ICD‐10‐BPA does not have a code for this condition. However, in the United States, ICD‐10‐CM Q55.64 (“hidden penis”) would capture this anomaly.

Finally, we compared our EUROCAT‐based ICD‐10‐CM code list to the NYS BDR since this was the most similar existing resource to EUROCAT. We added an additional column (“NYS minor”) to our code list indicating the New York State classification, as available. Codes not contained in the NYS BDR were assigned a value of missing (“.”) in the “NYS minor” column. Since the NYS BDR guidance does not clearly delineate major or minor classification for all codes, ambiguous codes were assigned a value (“1”) in the “NYS column” rather than render a decision for each. The principal investigator completed the initial classification, and the work was reviewed by a single reviewer.

## 3 Results

A final code list was created, containing 916 ICD‐10‐CM codes for 744 major and 172 minor malformations (Table 1; Supporting Information). The code list is in Microsoft Excel and can be imported into statistical analysis applications such as SAS or R. It contains ICD‐10‐CM codes, their corresponding descriptions, their major or minor classification and disease category according to EUROCAT, variables indicating anomalies caused by genetic or infectious diseases, and a toggle variable to indicate when a major anomaly code may include minor anomalies.

**TABLE 1:** Numbers and characteristics of anomalies in the EUROCAT translation to ICD‐10‐CM, by organ system.

Organ system	Total	Major	Minor	Genetic	Infectious	Toggle
Abdominal wall defects	5	5	0	0	0	0
Cardiovascular	20	17	3	0	0	0
Congenital heart defects^a^	85	80	5	0	0	1
Congenital anomalies of kidney and urinary tract (CAKUT)	61	56	5	1	0	0
Ear, face and neck anomalies	25	10	15	0	0	1
Eye anomalies	44	32	12	0	0	0
Gastro‐intestinal anomalies	66	56	10	0	0	5
Genetic disorders	146	139	7	146	0	0
Genital anomalies	110	64	46	0	0	0
Head	1	0	1	0	0	0
Limb anomalies	179	127	52	0	0	3
Nervous system anomalies	45	45	0	0	0	2
Oro‐facial clefts	16	15	1	0	0	0
Other anomalies/syndromes	38	37	1	0	5	1
Respiratory anomalies^b^	31	26	4	0	0	1
Skeletal	26	22	4	0	0	7
Skin	19	13	6	0	0	1
Total	917^b^	744	172	147	5	22

^a^ Two congenital heart defect codes (Q250 “Patent ductus arteriosus” and Q256 “Stenosis of pulmonary artery”) are classified as minor by EUROCAT if gestational age is less than 37 weeks. This information is indicated in the “Notes” column of the code list spreadsheet (Supporting Information).

^b^ One respiratory anomaly code was not classified in EUROCAT; we retained this code in the code list so that research teams may use it at their discretion.

## 4 Discussion

We adapted the EUROCAT Guide 1.5 into an ICD‐10‐CM code list for use in pregnancy studies using US data. To our knowledge this represents the first comprehensive classification system for major and minor congenital anomalies for use in pharmacoepidemiologic studies relying on US data.

In addition to EUROCAT, there are other coding and reporting guidelines available for reporting malformations to public health agencies [2, 3, 4]. These guidelines include the National Birth Defects Prevention Network (NBDPN) and New York State Department of Health Birth Defects Registry (NYS BDR). While all three of these standards include lists of diagnosis codes, we elected to translate the EUROCAT guide because it was the only system to list major and minor malformations in mutually exclusive code lists. In contrast, NYS BDR has many codes that could be classified as major or minor depending on the specific condition—information which is often not accessible in US healthcare data sources.

We compared our EUROCAT‐based ICD‐10‐CM code list to the NYS BDR since this was the most similar existing resource to EUROCAT. EUROCAT contained 32 codes not contained in the NYS BDR. A total of 34 codes were classified as major in our EUROCAT‐based code list that were classified as minor in the New York State guide, and a total of two codes were classified as major in our EUROCAT‐based code list that were classified as ambiguous (i.e., either major or minor) in the New York State guide. The “NYS minor” column does not represent a complete adaptation of the NYS BDR for use in ICD‐10 CM for two reasons. First, the NYS BDR includes 106 additional billable diagnosis codes under its “Other Selected Reportable Conditions” section that are not included in EUROCAT and are therefore not in our code list. These codes are exclusively for conditions falling outside of the ICD‐10 and ICD‐10‐CM “Q section” for congenital anomalies (e.g., P37.0, congenital tuberculosis). Second, the NYS BDR guidance does not clearly delineate major or minor classification for all codes. We have distinguished these ambiguous codes in the “NYS column” rather than render a decision for each one. The “NYS minor” column can be used to identify differences between the EUROCAT and NYS BDR.

Our adaptation of the EUROCAT guide will support United States studies of the relationship between drug exposures during pregnancy and congenital malformations but is not without its limitations. First, due to differences between coding systems, a direct translation was not possible for the entirety of the EUROCAT guide; we addressed this limitation through the inclusion of a toggle variable that allows researchers to take into account the uncertainty in major and minor classification for the small number of codes that could not be directly translated. Second, as new ICD‐10‐CM codes become available or as the EUROCAT Guide is updated, further updates to this list will be needed. Third, the accuracy of codes is beyond the scope of this work. A comparison of the EUROCAT registry to electronic hospital databases for birth years 2010–2014 showed that electronic health care data did not capture all congenital anomalies reported to the registry and that validity of health care data to ascertain congenital anomalies varied by specific anomaly (e.g., cleft palate, spina bifida), with some anomalies more completely captured than others [9]. Uncertainties in coding accuracy and completeness can be addressed in US database studies using coding algorithms and medical record validation.

## Ethics Statement

This study did not involve human subjects.

## Conflicts of Interest

Krista Schroeder and Maryline Le Noan‐Lainé are employees and stockholders of Eli Lilly and Company.

## Supporting information


**Appendix A.** EUROCAT_Codelist.
